# Classification of Program Types and Cost Prediction of Integrated Care for the Elderly

**DOI:** 10.3389/fpubh.2022.818811

**Published:** 2022-04-28

**Authors:** Byunggeor Moon

**Affiliations:** School of Global Leader and Department of Public Administration and Graduate School of Governance, Sungkyunkwan University, Seoul, South Korea

**Keywords:** health policy and practice, community care, health insurance, financial integration, elderly

## Abstract

This paper analyzes the types of community-based integrated care programs based on the needs of consumers and estimates future costs through data on consumer-oriented integrated care for the elderly in the local community operated by Korea, which is experiencing rapid aging. By analyzing the types of programs and the composition of budget items, we check the composition of the program from the consumer side of the integrated care program in the local community and predict the national budget for each item to operate it. Through this, policy implications for business operation and implications for sustainable financial management are derived.

## Introduction

In a community-centered integrated care program, complex programs are run organically, making it difficult to apply a uniform unit price to the total program. In the case of integrated care for the elderly, there is a lot of discussion about the importance of responding to various demand groups, but there is not much analysis on what types of programs are in demand.

There is considerable discussion around the effectiveness of integrated care for the elderly (1, 2). Studies have used reduction of admission to medical institutions, satisfaction of program targets through questionnaires, and cost reduction as indicators of program effectiveness. However, the literature shows mixed results about the effectiveness of the business of integrated care for the elderly.

Furthermore, there have also been analyses of the characteristics of each country and the effectiveness of programs on the effectiveness of integrated care (3). Goodwin et al. analyze the program in the United States, where incentives are provided using private resources (4). There have also been papers that analyze programs in the UK, Germany, Australia, and Switzerland, where integrated care for the elderly is operated at the central government level (5–8). Johri et al. find quantitative effects such as a decrease in the number of patients and institutional admissions (9), but their study does not indicate a unique difference compared to other countries. What appears to be important in the effectiveness of each country's integrated care program for local communities is improving the level of coordination between the existing fragmented programs, and this requires an overarching system (10, 11).

In addition, there have been studies on the taxonomy of integrated care. A representative study derives key features of integrated care work are through existing literature analysis and the Rainbow Model and provides a classification based on the key features (12).

An important factor in operating a community-based integrated care program is the analysis of the needs of the program beneficiaries and a structural approach to the associated costs. However, not much research has been done on this topic. Rather, the existing literature has focused on providing a framework for cost analysis or analyzing the economic effects of integrated care (13, 14).

Korea is experiencing the highest rate of population aging among OECD countries and is making mid- to long-term plans to operate integrated care programs subject to limited financial resources. One such plan was for the Korean government to decentralize its integrated care program and to conduct a pilot program for integrated care for the elderly targeting 13 local governments. The main feature of this program is that it was constructed by reflecting on the needs of the local community seniors, and the central government allocated financial resources in a top-down manner. The characteristics of this program provides us with information on the demand of the local governments, and based on this, it is possible to estimate the expected cost assuming that the integrated care program for the elderly will be expanded nationwide.

Our research contributes to the existing studies and findings in several aspects. First, given the complexity of the integrated care program for the elderly, our data helps confirm the needs of actual consumers on a per program basis. In addition, our study presents a framework for conducting effective analysis of managing integrated care programs by classifying various programs into several major categories. Moreover, by analyzing each program by budget item, our study provides a basis for finding efficient management methods in terms of budget management. Finally, through long-term cost forecasting, we identify long-term fiscal demand forecasts and structural factors that can influence fiscal demand.

Our paper is organized as follows. Section A Pilot Program of Integrated Care for the Elderly in the Local Community in Korea describes the features of the pilot program of integrated care for the elderly in Korea, as well as the budget for each local government and the method of classifying major programs, and based on this, 4 categories are formed. In section Mid- to Long-Term Cost Estimation for Integrated Care for Elderly, we forecast the financial need according to the scenario of future growth of the elderly population and business expansion based on the financial requirements of the pilot program in 2020. Section Conclusion and Policy Implications draws policy implications and provides the concluding remarks.

## A Pilot Program Of Integrated Care For The Elderly In The Local Community In Korea

The data we use is based on internal data on the contents and the budget of a pilot program of integrated care for the elderly in 13 local governments in 2020. The program is operated as follows. First, individual local governments submit their wishes to the central government about whether they want to run an integrated care pilot program for the elderly. Next, the local government to operate the pilot program is selected based on the proportion of the elderly population and whether existing programs are being operated. The local governments selected in this way design their programs and calculate their budgets by reflecting their needs. The central government then makes minimal interventions to adjust excessive unit prices, and the budget is allocated in a top-down manner.

After budget allocation, the local government operates the program by reflecting on the needs of the elderly. During this process, the requests of the consumers are met as much as possible, except in cases where it is difficult to fulfill the requests due to legal issues or excessive demands that exceed the budget range. After that, the central government evaluates individual projects supported through the program.

The fact that these programs are decentralized in the above manner makes it possible to select and operate projects that reflect the needs of individual local governments rather than having a central government-centered project selection and operation. Because of this, we are able to analyze the types of programs required for integrated care from the perspective of actual consumers.

We now describe the social characteristics and budget allocation status of the 13 local governments selected for analysis. The population composition of the 13 local governments operating the program is as follows.

As shown by Table 1, the local governments that the program targets have significant elderly populations, and on average, about 14% of the population is 65 years or older.

**Table 1:** Characteristics of the composition of the local population in the operation of integrated care for the elderly in the local community.

**Name of Local Government**	**Total population**	**Number of senior citizens aged 65 and over**	**Proportion of elderly population in the area (%)**
Gwangju Seo-gu	304,775	43,487	14.27
Bucheon	864,895	117,552	13.59
Cheonan	679,618	74,119	10.91
Jeonju	663,210	100,423	15.14
Gimhae	562,728	63,115	11.22
Busan Jin-gu	359,848	71,363	19.83
Busan Buk-gu	291,190	50,203	17.24
Suncheon	284,557	45,326	15.93
Seogwipo	186,446	35,872	19.24
Jincheon-gun	89,422	14,232	15.92
Cheongyang-gun	31,914	11,329	35.50
Namyangju	717,118	101,576	14.16
Ansan	722,051	77,386	10.72
Total	7,292,213	805,983	13.58

The budget provided to the local governments for the program is as follows.

As shown by Table 2, a total of KRW 250 billion was used annually for the community-centered integrated care for the elderly pilot program.

**Table 2:** Budget status by region for the pilot program for integrated care for the elderly in the local community.

**Name of Local Government**	**Unit: KRW 1,000 (Approximately 1$)**
Gwangju Seo-gu	2,141,900
Bucheon	1,880,232
Cheonan	2,130,500
Jeonju	1,793,700
Gimhae	2,130,800
Busan Jin-gu	1,990,500
Busan Buk-gu	1,890,500
Suncheon	2,135,500
Seogwipo	2,130,000
Jincheon-gun	2,050,500
Cheongyang-gun	1,389,000
Namyangju	1,793,500
Ansan	2,082,500
Total	25,746,132

Our study classifies the types of pilot programs in 13 local governments based on the program composition and purpose of each project and confirms the proportion of each budget item. First, as a criterion for classifying the program type, we classify the purpose of the program description by type. In the case that a single program has a complex nature, we classify the type based on the program that occupies the highest proportion in the budget.

If the types of projects are classified based on this method, they can be broadly classified into housing support, health and medical support, daily care support, and common operating expenses. The major programs in each category are shown in Table 3 below.

**Table 3:** Classification of major program types of the pilot programs for elderly.

**Housing support**	**Health and medical support**
Residential Environment Improvement Home Repair	Provision of home-based medical services
Care safe housing infrastructure installation Operation facility installation	Disease Prevention and Visiting Health Care Visiting Nursing
Care Security Housing Residential Infrastructure Installation Operation Rental Deposit	Disease prevention and visiting health management Chronic disease management
Housing-related expenses support rental deposit	Visiting health management program
Care Safe (health related) housing infrastructure installation	Health management programs such as base-type senior citizens
Care Security Housing Infrastructure Installation	Visiting drug abuse prevention education and guidance
Initial installation of smart home utilization equipment	Rehabilitation Rehabilitation Exercise Therapy
Smart Home Utilization Communication Fee	Support for quasi-drugs
	Protective equipment support
**Daily care support**	**Miscellaneous program operation**
Meal support	Common operating expenses
Housekeeping support	Case conference support
Mobility support	Personalized discretionary support
Nursing service	
Care support service	
Emergency care support	
Bath support	
Hairdressing support	
Daily necessities support	
Emotional development and healing support	
Emotional development and healing support, social adaptation training, etc.	
Support for caring families	
Nurturing care workers	
Safety and Human Rights Education	

Thus, the demand for integrated care for the elderly can be divided into housing services for integrated care for the elderly, health care support before medical treatment, and support and operating costs for daily living.

Table 4 shows the reclassification of project budgets by municipalities based on the classification of these program types.

**Table 4:** Budget status by program type and region.

**Municipality name**	**Housing support**	**Health and medical support**	**Daily care support**	**Miscellaneous program operation**
Gwangju Seo-gu	656,800	309,400	810,000	365,700
Bucheon	400,000	249,000	420,676	810,556
Cheonan	315,000	547,590	1,110,000	157,910
Jeonju	395,000	515,800	502,500	380,400
Gimhae	437,000	333,600	1,229,200	131,000
Busan Jin-gu	836,000	207,000	539,000	408,500
Busan Buk-gu	440,000	194,400	860,000	396,100
Busanjin-gu	836,000	207,000	539,000	408,500
Suncheon	775,000	91,600	993,997	274,903
Seogwipo	784,000	100,000	779,200	466,800
Jincheon-gun	610,000	443,600	693,300	303,600
Cheongyang-gun	700,000	24,000	552,000	113,000
Namyangju	400,000	263,000	987,200	143,300
Ansan	900,000	100,000	764,000	318,500
Total	7,648,800	3,585,990	10,241,073	4,270,269

We now break these programs down again by budget category for analysis. We use the budget item code that is utilized when composing the budget for this type of program and divide the program into labor cost, operating cost, and business cost. The reason for this classification is to classify the programs by budget items to increase the effectiveness of budget or public finance management. At the same time, this classification lays the basis for analysis of future changes in budget items. The composition of major program types by budget item is shown in Table 5 below.

**Table 5:** Composition of budget for integrated care for the elderly in local communities by type.

**Program type**	**Budget item**	**Unit: KRW 1,000**
Housing support	Labor costs	1,391,850
	Operating expenses	334,272
	Business expenses	5,968,883
	Total	7,695,005
Health and medical support	Labor costs	2,850,785
	Operating expenses	398,445
	Business expenses	188,021
	Total	3,437,251
Daily care support	Labor costs	5,159,839
	Operating expenses	996,467
	Business expenses	4,160,075
	Total	10,316,380
Miscellaneous Program Operation	Labor costs	913,236
	Operating expenses	3,225,735
	Business expenses	158,525
	Total	4,297,496

The following describes the operation methods of the 13 local governments for each classification based on the number of services provided and the unit price. In the case of housing support, we are promoting facility improvement programs for about 1,671 households with an average program cost of KRW 3,300 per household, mainly for the improvement of care and safety housing facilities. For residential infrastructure installation and operation management and smart home installation and operation, an annual wage of 30 million won per person is provided, and an average of 18 people are in charge of related program operation. As we can see from the related contents, the proportion of related program costs is high in the case of integrated care, because it focuses on the organic connection process of existing program systems.

In the case of health and medical support, an average of 2,642 medical treatment support services are provided for oriental doctors, doctors, dentists, and assistants by setting an average labor cost of KRW 193,000, KRW 181,000, KRW 75,000, and KRW 40,000 per local government, respectively. Also, for rehabilitation and exercise support, an average of 515 cases and 1,168 cases are provided by applying an average labor cost per session of KRW 504,000 for rehabilitation and KRW 80,000 for exercise. In addition, for the medication support program, an average of KRW 86,000 per session is provided to related personnel, providing an average of 2,704 services.

In the case of daily care support, the average price per meal is set at KRW 5,990 per meal to provide lunchboxes or ingredients to an average of 594 people. A total of 161,805 laundry and cleaning services are provided based on the labor cost allowance. In addition, it provides an average of 27,743 opportunities to use the service by subsidizing expenses such as taxis and other means of transportation for an average of KRW 390,000 per month.

For miscellaneous operation, funds are used for research services to create a care base and for supporting local care meetings and case meetings. In the case of local care meetings, an average of 95,000 won per local government is paid to about 20 meeting attendees and held 5 times a year on average. In order to revitalize local community care, an average of 1.2 studies is being conducted, and about KRW 23 million is paid per case. The budget is also used for publicity expenses and production of promotional materials.

## Mid- To Long-Term Cost Estimation For Integrated Care For Elderly

This section estimates mid- to long-term costs based on the content of the pilot program. In estimating the cost, we assume that the operation details and composition of the integrated care project pilot program of 13 local governments will remain as the basis for future operations. This is because the program has already reflected the needs of consumers. Based on this assumption, we estimate the cost if the program were to expand over the next 5 years while taking into consideration the coverage of the elderly population currently covered by the pilot program. The detailed calculation method and contents are described in the following section.

### Changes in Social and Structural Characteristics Underlying Cost Estimation

In order to estimate the cost under the scenario where there is an expansion of the range of users of the pilot program, we first consider future population changes and the estimation of the elderly population. To do this, we apply the forecast for the population aged 65 and over for 5 years starting 2022 using the 2019 forecast of the Korean National Statistical Office, which is the most recent forecast on overall population and elderly population of Korea. Table 6 shows the forecast.

**Table 6:** Future Korean population and elderly population estimation.

**Year**	**Total population**			
	**Total**	**0 to 14**	**15–64**	**65+**
2020	51,781	6,297	37,358	8,125
2021	51,822	6,152	37,133	8,537
2022	51,846	5,985	36,887	8,975
2023	51,868	5,801	36,620	9,447
2024	51,888	5,661	36,282	9,945
2025	51,905	5,541	35,853	10,511
2026	51,920	5,395	35,411	11,114

The total population at the time of the study is expected to increase from 51.8 million to 51.9 million, while the elderly population over 65 is expected to increase significantly from 8,975 thousand to 11.1 million. As the community-based integrated care program targets the entire elderly population over 65, the expansion of related programs will be discussed based on the statistics above.

In our study, we assume that costs and prices will increase 1.4% per annum, which is based on the inflation rate predicted by the National Statistical Office of Korea for the next 10 years, and that labor costs will increase 2.9% per annum, based on the 2020 supplementary guidelines for budgeting for local investment institutions.

Based on the 2020 budget of the pilot program for integrated care for the elderly in the local community, the mid- to long-term budget for the program is estimated as follows. First, we confirmed what percentage of the elderly population in the area is targeted by the pilot program. Next, we predict the scope of the program based on the population estimate and the annual expansion plan of the program. The number of elderly people in the pilot program community is 805,983 and the number of elderly people who are provided integrated care through the pilot program is 14,337, which is about 1.78% of the total elderly population. In the future, the local government that will operate the program will increase the number of the relevant cities, counties, and town by 20% per year (~40 cities, counties, and town) for all 223 cities, counties, and town, and finally, in 2025, the local government is expected to provide priority service at the level of the current pilot program. The number of beneficiaries of the program is predicted as follows under the premise that the service level currently in charge of the pilot program is provided in connection with the future forecast for the elderly population. Tables 7, 10 show the estimation following the ways stated above.

**Table 7:** Estimation of recipients of integrated care for the elderly in the local community.

**Year**	**National elderly population**	**Program target scope (Based on the number of cities, counties and districts)**	**Number of subjects**	**Elderly population coverage (%)**
2022	8,975,000	0.26	37,749	0.46
2023	9,447,000	0.41	62,658	0.73
2024	9,945,000	0.56	90,093	1.00
2025	10,511,000	One	170,036	1.78
2026	11,114,000	One	179,791	1.78

Based on these forecasts, the estimates for each program type and budget item given the expansion of the target of integrated care and the increase in cost are shown in Table 8 below.

**Table 8:** Estimated budget for each program type and item for the integrated care for the elderly in the local community.

**Housing support**	**Labor costs**	**Operating expenses**	**Business expenses**	**Total**
2022	1,658,095	453,181	20,367,902	22,479,177
2023	2,752,195	752,214	33,807,749	37,312,158
2024	3,957,257	1,081,575	48,610,635	53,649,467
2025	7,468,709	2,041,304	91,745,024	101,255,037
2026	7,897,177	2,158,411	97,008,296	107,063,883
**Health and medical support**
2022	8,327,909	1,163,964	549,261	10,041,134
2023	14,223,987	1,959,059	924,457	17,107,502
2024	21,045,137	2,856,278	1,347,844	25,249,259
2025	40,871,290	5,466,251	2,579,461	48,917,002
2026	44,469,278	5,860,760	2,765,625	53,095,662
**Daily support**
2022	15,073,275	2,910,947	12,152,695	30,136,917
2023	25,745,004	4,899,392	20,454,108	51,098,503
2024	38,091,088	7,143,239	29,821,778	75,056,105
2025	73,975,852	13,670,499	57,071,950	144,718,300
2026	80,488,105	14,657,121	61,190,926	156,336,152
**Miscellaneous program operation**
2022	2,667,806	9,423,238	463,095	12,554,139
2023	4,556,587	15,860,178	779,432	21,196,197
2024	6,741,710	23,123,899	1,136,400	31,002,008
2025	13,092,924	44,253,766	2,174,805	59,521,495
2026	14,245,522	47,447,633	2,331,764	64,024,919

From the perspective of the entire program, the cost of the expansion scenario of the integrated care program for the elderly in the community is estimated to be as follows in Table 9. If the current coverage for the elderly population is applied and the program is expanded step by step based on the performance and data of the pilot program, we estimate that it will cost about 1.11 trillion won over the next 5 years.

**Table 9:** Estimated cost by item of integrated care for the elderly in the local community (overall).

**Year**	**Labor costs**	**Operating expenses**	**Business expenses**	**Total**
2022	22,479,177	10,041,134	30,136,917	75,211,367
2023	37,935,762	17,107,502	51,098,503	127,337,964
2024	55,459,546	25,249,259	75,056,105	186,766,917
2025	106,427,562	48,917,002	144,718,300	359,584,359
2026	114,425,105	53,095,662	156,336,152	387,881,838
Total	336,727,152	154,410,559	457,345,977	1,136,782,446
	29.6%	13.6%	40.2%	

**Table 10:** Estimation of recipients of the integrated care for the elderly in the local community according to the expansion of the target range.

**Year**	**Elderly population**	**Program target scope**	**Beneficiaries**	**Elderly population coverage**
		**(Based on the number of cities, counties and districts)**	
2022	8,975,000	0.26	41,509	1.8%
2023	9,447,000	0.41	87,149	2.3%
2024	9,945,000	0.56	155,938	2.8%
2025	10,511,000	1.0	367,885	3.5%
2026	11,114,000	1.0	450,117	4.1%

We also predict that the proportion of business expenses will be higher than other items in terms of establishing a system of integrated care programs. The expected totals and unit prices for each program in detail are shown in the Appendix 1.

### Exploring the Possibility of Cost Change

The cost estimate of the community-centered integrated care program presented in the previous section is based on the number of elderly people in the area and the number of service recipients receiving services through the program based on the results of the pilot program currently being implemented. We calculated the cost conservatively, assuming a nationwide operation of the related program in the future. Therefore, in the expansion and operation of related programs in the future, the following should be considered, and changes in costs can be expected accordingly.

#### Changes in the Size of Beneficiaries

In the previous section, the proportion of service recipients among the elderly population in the region appeared to be about 1.78% in the case of the regional integrated care pilot program, and we estimated the cost of the nationwide expansion of the program in stages from 2022 to 2026.

However, the number of the potential beneficiaries of the project varies depending on the type and characteristics of the target(s). Given the number of people in the blind spot of welfare, the number of beneficiaries may be expanded from 179,000 to about 450,000 in 2026 (15).

Assuming such a change in demand, the coverage due to the expansion of the service target will increase to 4.1% in 2026, and the related costs are will increase to about KRW 2.1 trillion as follows in Table 11.

**Table 11:** Estimated increase in cost by item of integrated care program due to expansion of program targets.

**Year**	**Labor costs**	**Operating expenses**	**Business expenses**	**Sum**
2022	22,479,177	10,041,134	30,136,917	75,211,367
2023	48564165.9	21376709.87	64158900.4	160,826,457
2024	89417156.4	38785408.67	116408426.4	293,103,264
2025	217068273	92782579.68	278472615.6	704,326,839
2026	273290831	115111240.6	345488649.9	877,810,970
Sum	650,819,603	278,097,073	834,665,509	2,111,278,896
	30.8%	13.2%	39.5%	

#### Changes in Cost Composition Due to the Advancement of Program Operations

We confirm that the proportion of labor and operating expenses rather than the program cost will increase in order to actually operate the integrated care program after establishing the program through phased expansion over the next 5 years. In this case, we predict that in the future, the cost of personnel expenses and labor costs will rise compared to the inflation, and additional costs will be required in the field of training and composition of related experts, and the proportion of program expenses will be adjusted.

## Conclusion And Policy Implications

The complexity and diversity of the integrated care for the elderly in the community are important issues. For the global aging population, integrated care is becoming a major alternative to conventional care provision. Based on the data of Korea's consumer-centered integrated care pilot program, this paper analyzes program types based on consumer needs and classifies them by budget item, contributing to program operation and financial planning at the same time.

We derive the following policy implications. First, program operation centered on the needs of consumers is necessary in the future. Efforts to save costs by deriving common areas of operation through analysis of program types are also important. In addition, by analyzing the types of expenses as well as the contents of the program, it is possible to derive the items of expenses that can be increased in the future and to confirm that it is necessary to prepare for them.

For countries experiencing rapid aging, community care is becoming an alternative to improving the health and well-being of the elderly. Important factors for the success of community care are beneficiary satisfaction and sustainable financial operations. This study presents the service needs and cost estimates of beneficiaries in studies on the effectiveness and cost of existing community care. Future research may analyze cost-saving factors of community care programs for the elderly in terms of sustainable financial operation and analyze various types of services.

## Author's Note

This research was based on the results of the policy research project of the Ministry of Health and Welfare of Korea and may differ from the official opinion of the institution.

## Data Availability Statement

The original contributions presented in the study are included in the article/Supplementary Material, further inquiries can be directed to the corresponding author/s.

## Author Contributions

The author confirms being the sole contributor of this work and has approved it for publication.

## Funding

This research was supported by the SungKyunKwan University, the BK21 FOUR (Graduate School Innovation) funded by the Ministry of Education (MOE, Korea), and National Research Foundation of Kore (NRF).

## Conflict of Interest

The author declares that the research was conducted in the absence of any commercial or financial relationships that could be construed as a potential conflict of interest.

## Publisher's Note

All claims expressed in this article are solely those of the authors and do not necessarily represent those of their affiliated organizations, or those of the publisher, the editors and the reviewers. Any product that may be evaluated in this article, or claim that may be made by its manufacturer, is not guaranteed or endorsed by the publisher.
