# Supplementary material for: Classification of Program Types and Cost Prediction of Integrated Care for the Elderly
Source: Front Public Health. 2022 Apr 28;10:818811. doi: 10.3389/fpubh.2022.818811 (PMC9097149; doi:10.3389/fpubh.2022.818811)
Supplement: Supplementary file 1 [file Table_1.pdf]

Appendix-1

As of 2026 (if nationalized)

Health care	Major expenditure details.	Expected total (KRW 1,000)	Estimated unit price (KRW 1,000)
Labor costs	Home visit type medical service provision _ Oriental Medicine	6,673,704	217
	Home visit type medical service provision _ Medicine	10,542,072	203
	Home visit type medical service provision _ Dentistry	2,498,076	84
	Home visit type medical service provision _ Assistant staff	4,229,430	45
	Disease prevention and visit health management _ Visiting nursing (nursing)	1,062,079	67
	Disease prevention and visiting health management _ Visiting nursing (assistant staff)	1,161,748	34
	Disease prevention and visiting health management _ Visiting nursing (operating personnel)	276,788	561
	Visiting health management program	5,369,118	31,607
	Base-type (Senior-center, etc.) health management program	863,884	30,513
	Visiting type of drug abuse prevention education and guidance	3,719,499	97
	Rehabilitation treatment _ Rehabilitation , exercise therapy (rehabilitation)	4,120,397	565
	Rehabilitation treatment _ Rehabilitation , exercise therapy (exercise)	3,339,233	202
	Rehabilitation treatment _ Rehabilitation , exercise therapy (regional linkage)	41,899	34

	Rehabilitation treatment _ Rehabilitation , exercise therapy (operating personnel)	571,352	40,361
Operating expenses	Home visit type medical service provision _ Medicine , dentistry, oriental medicine	1,483,829	529
	Disease prevention and visit health management _ Chronic disease management	29,931	211
	Visiting health management program	3,347,783	7
	Base type (Senior-dang, etc.) health management program	128,224	755
	Visit-based drug abuse prevention education and guidance	380,124	2
	Rehabilitation treatment _ Rehabilitation , exercise therapy	490,868	533
Business expenses	Disease prevention and visit health management _ Chronic disease management	140,676	108
	Rehabilitation treatment _ Rehabilitation , exercise therapy	643,517	190
	Drug (quasi -drug) support	299,310	2,889
	Equipment support	1,682,122	3,788

As of 2026 (if nationalized)

Daily care	Major expenditure details.	Expected total (KRW 1,000)	Estimated unit price (KRW 1,000)

Labor costs	Meal support	11,500,317	33,634
	housekeeping support	40,553,281	24
	Mobility support	9,854,486	16
	Nursing service	316,045	112
	Care support service	3,813,477	387
	Emergency care support	8,330,945	17
	bath support	1,697,161	73
	hairdressing support	63,209	19
	Emotional development and healing support _ Safety check	1,074,553	33,634
	Support for emotional development and healing _ Social adaptation training, etc.	2,003,520	33,634
	Support for caring families	214,459	11
	Nurturing care workers	1,066,652	33,634
Operating expenses	Daily necessities support	2,309,622	910
	Emotional development and healing support _ Safety check	101,325	5
	Support for emotional development and healing _ Social adaptation training, etc.	244,373	53
	Nurturing care workers	2,415,418	118
	Meal support	7,955,757	78
	housekeeping support	379,079	2,241
	Mobility support	1,147,241	10
	Care support service	104,306	41
Business expenses	Meal support	41,178,817	6
	Mobility support	9,053,645	15
	Nursing service	134,107	190
	Care support service	447,024	93
	Emotional development and healing support _ Safety check	3,725,197	826

	Support for emotional development and healing _ Social adaptation training, etc.	83,444	15
	Support for caring families	2,241,504	177
	Nurturing care workers	4,252,684	36
	Safety and Human Rights Education	74,504	53

As of 2026 (if nationalized)

Housing Support	Major expenditure details.	Expected total (KRW 1,000)	Estimated unit price (KRW 1,000)
Labor costs	Residential environment improvement _ house repair	11,407,893	33,634
	Care Security Housing Residential Infrastructure Installation Operation Labor Cost	6,418,514	45,855
	Smart home (using IoT) _ initial equipment installation	3,885,006	34,694
Operating expenses	Residential environment improvement _ house repair	1,493,474	529
	Residential environment improvement _ house repair (spare material cost)	29,596	529
	Care safe housing housing infrastructure installation and operation program operation	1,346,613	2004
	Smart home (using IoT) _ initial equipment installation	809,448	1.368
	Smart home (using IoT) _ Communication cost (use fee)	1,237,700	20
Business expenses	Residential environment improvement _ house repair	70,129,591	5,110
	Residential infrastructure installation operation facility installation for care safe housing	6,215,138	34,155

	Care Security Housing Residential Infrastructure Installation and Operation Rental Deposit	7,029,025	125,541
	Care safe housing infrastructure installation and operation program operation	295,959	529
	Housing-related expenses support _ rental deposit	739,897	5,606
	Smart home (using IoT) _ initial equipment installation	3,387,250	1,283

As of 2026 (if nationalized)

Miscellaneous Operation	Major expenditure details.	Expected total (KRW 1,000)	Estimated unit price (KRW 1,000)
Labor costs	Consolidated operating expenses	7,856,959	78
	Case conference support	6,388,564	33,634
Operating expenses	Consolidated operating expenses	43,716,139	260,175
	Case conference support	3,731,494	2,208
Business expenses	Case conference support	1,443,591	8,591
	Personalized discretionary support	888,173	6,343
